# Barriers to Point-of-Care Ultrasound Utilization Among Emergency Medicine Residents in Riyadh, Saudi Arabia

**DOI:** 10.7759/cureus.65765

**Published:** 2024-07-30

**Authors:** Fahad Abuguyan, Naief W Almasry, Ali A Alzahrani

**Affiliations:** 1 Emergency Medicine, College of Medicine, King Saud University, Riyadh, SAU; 2 Emergency Medicine, King Khalid University Hospital, Riyadh, SAU

**Keywords:** bedside ultrasonography, emergency departments, emergency residency, barriers, point-of-care-ultrasound

## Abstract

Background: Point-of-care ultrasound (POCUS) is increasingly recognized as a valuable diagnostic tool in emergency medicine (EM). This study aimed to identify barriers to POCUS utilization among EM residents in the Riyadh region.

Materials and methods: An observational cross-sectional study was conducted among 116 EM residents from various training centers in Riyadh. Data were collected through self-administered questionnaires assessing demographics, ultrasound (US) training, perceived barriers, and facilitators to POCUS usage. Statistical analysis included descriptive statistics, multiple response dichotomy analysis, and multivariable linear regression.

Results: The majority of residents had completed US training and recognized the importance of POCUS in emergency settings. However, significant barriers were identified, including time constraints and logistical challenges. Multivariable regression analysis revealed associations between residents' training status, beliefs in incentives, anticipated POCUS use, and perceived barriers.

Conclusion: This study highlights the importance of addressing barriers to POCUS integration into residency programs. Efforts should focus on optimizing training, addressing workflow challenges, and enhancing residents' confidence in POCUS utilization. Targeted interventions tailored to specific clinical contexts may facilitate greater acceptance and integration of POCUS into routine practice.

## Introduction

The landscape of emergency medicine (EM) is characterized by the urgent need for rapid decision-making in response to patients' critical conditions, often necessitating swift diagnostic evaluations [1,2]. Traditionally, clinicians rely on a combination of history-taking, bedside assessments, and laboratory investigations to inform clinical decisions [3]. Ultrasound (US) has emerged as a pivotal diagnostic tool in EM, offering rapid assessment capabilities that aid in early disease detection, enhance diagnostic accuracy, and mitigate procedural complications when utilized effectively [4].

Point-of-care ultrasound (POCUS) has particularly gained prominence in emergency departments (EDs) globally, being increasingly integrated into clinical practice during shifts [5]. In addition, benefits extend beyond rapid assessment capabilities, enhancing diagnostic accuracy and mitigating procedural complications [6]. POCUS allows for real-time visualization, enabling immediate feedback and adjustments during procedures, thereby reducing errors and improving patient outcomes. Moreover, its portability facilitates bedside evaluation, eliminating the need for patient transportation to imaging facilities, which can be crucial in time-sensitive situations. Additionally, POCUS is non-invasive and radiation-free, making it safe for repeated use, particularly in vulnerable populations such as pregnant women and pediatric patients. Its versatility also extends to various medical specialties beyond EM, including critical care, internal medicine, and primary care, where it aids in prompt diagnosis and decision-making [7-10].

Recognizing the potential of POCUS to significantly improve patient outcomes, medical training programs worldwide have increasingly incorporated POCUS education into their curricula [11-13]. Accrediting bodies, such as the Accreditation Council for Graduate Medical Education (ACGME), have underscored the importance of POCUS skills by integrating them into training milestones for EM residents [14,15]. Despite its recognized benefits, studies have revealed various obstacles hindering its widespread adoption among emergency physicians. These barriers range from time constraints and knowledge gaps to insufficient training opportunities and reluctance or opposition encountered when trying to implement new practices, procedures, or technologies within the healthcare industry. Physicians often face challenges in allocating time for POCUS amidst the fast-paced nature of EDs, where they juggle multiple responsibilities simultaneously. Additionally, while POCUS education is becoming more common, some physicians may still lack comprehensive training or experience in utilizing this technology effectively. Furthermore, the availability of POCUS equipment and resources may vary across different healthcare settings, posing logistical challenges to its implementation [7,16-21].

Studies conducted in various healthcare contexts have consistently highlighted the importance of overcoming these obstacles to optimize the integration of POCUS into clinical practice [22]. However, the applicability of findings from international studies to the Saudi Arabian context, or any specific context, remains an important consideration, as cultural, organizational, and resource-related factors can influence the adoption of POCUS practices [23]. Therefore, tailored strategies that account for the unique challenges and needs of emergency physicians in Saudi Arabia, as well as other regions, are essential for maximizing the benefits of POCUS and improving patient care [24].

In light of the aforementioned gaps in knowledge and the growing importance of POCUS in EM, this study aims to identify the barriers to POCUS use among EM residents in Riyadh, Saudi Arabia. By elucidating these barriers, we seek to inform targeted interventions aimed at optimizing POCUS utilization and enhancing patient care outcomes in the ED setting.

## Materials and methods

Study design and participants

This is an observational cross-sectional study conducted over a period of six months from September 2023 to February 2024 to identify the barriers to POCUS use among EM residents in Riyadh, Saudi Arabia. Included in the study were 116 EM residents from various training centers in Riyadh, including King Khalid University Hospital (KKUH), King Saud Medical City (KSMC), Prince Mohammed bin Abdulaziz Hospital (PMAH), King Fahad Medical City (KFMC), Prince Sultan Military Medical City (PSMMC), National Guard Health Affairs (NGHA), King Abdulaziz University Hospital (KAAUH), King Faisal Specialist Hospital & Research Centre (KFSHRC), Security Forces Hospital (SFH), and Habib medical group hospital (HMG). These centers collectively serve a diverse patient population with varying acuity levels. 

Inclusion/exclusion criteria

All current EM residents in the Riyadh region were included in the study. Rotators from EM programs outside the Riyadh region and non-resident participants were excluded from the study.

Sample size

In the study, the convenience sampling technique was employed. The estimated total population of Riyadh's residents is around 368 individuals. Utilizing the Krejcie and Morgan sample size calculation formula, a sample size of approximately 189 was deemed appropriate. In an attempt to maximize the response rate, questionnaires were distributed to all residents.

Procedure and data collection

Data was collected through a self-administered questionnaire to EM residents in Riyadh, Saudi Arabia. The questionnaire was written in English and was distributed via email and WhatsApp using SurveyMonkey. Participants were asked to complete the questionnaire anonymously. Participation was completely voluntary, and data collection was entirely anonymous. After they were instructed about the nature and purpose of the survey, all respondents provided informed consent and were given the option to withdraw at any time. The questionnaire's development was guided by the study's objectives and informed by a thorough literature review. It underwent content validity testing, leading to adjustments in the initial draft. The Cronbach's alpha test of internal consistency indicated that the questionnaire was reliably comprehended by the physicians, with a Cronbach's alpha value of 0.899. The questionnaire comprised several sections and aimed at gathering comprehensive data regarding the attitudes, experiences, and perceptions of EM residents toward POCUS. The first section focused on demographic information, including the respondents' level of training and completion status of the US rotation. Following this, participants were asked to anticipate their usage of POCUS in various clinical indications as attending physicians. Subsequently, their experiences with POCUS were assessed, specifically in terms of frequency of use during shifts. The questionnaire then delved into the residents' attitudes toward POCUS, prompting them to express their agreement or disagreement with statements regarding the importance of POCUS in their training and future practice. Additionally, respondents were asked to rate the perceived barriers hindering their performance of POCUS, encompassing factors such as time constraints, equipment availability, and scope of practice concerns. Finally, the questionnaire explored potential facilitators to enhance POCUS usage, including incentives, support from attendings, and clarity in guidelines and documentation. Through these comprehensive sections, the questionnaire aimed to capture a holistic understanding of the residents' perspectives on POCUS utilization in the ED setting.

Ethical considerations

This study was performed in line with the principles of the Declaration of Helsinki. The questionnaire and methodology for this study were approved by the Institutional Review Board Committee of King Saud University, College of Medicine (Ref. No. 23/0292/IRB). Informed consent was obtained from all individual participants included in the study.

Statistical analysis

Statistical analyses of the collected data were performed using IBM SPSS Statistics for Windows, Version 28 (Released 2021; IBM Corp., Armonk, New York, United States). The mean and standard deviation were employed to describe continuously measured variables, while frequency and percentages were utilized for categorically measured variables. Multiple response dichotomy analysis was employed to describe variables measured with more than one option. Histograms and the Kolmogorov-Smirnov statistical normality test were utilized to assess the statistical normality assumption for metric variables. Cronbach's alpha test was employed to assess the internal consistency of the questionnaire. Multivariable linear regression analysis was conducted to assess predictors for physicians' mean perceived barrier to using POCUS machines in their daily clinical practice. The association between predictor variables and the analyzed outcome in the linear regression was expressed as beta coefficients with their corresponding 95% confidence intervals. The alpha significance level was set at 0.050.

## Results

Descriptive analysis of the residents’ professional characteristics and US training levels is presented in Table 1. One hundred and sixteen EM residents participated in the study and completed the questionnaire, with a response rate of 61.3%. The findings showed that 34.5% were in Residency Level 1, 29.3% were in Level 2, 19% were in Level 3, and 17.2% were in Level 4. Regarding the US training, the majority of the residents (83.6%) had completed it, while 16.4% did not yet.

**Table 1:** Descriptive analysis of the residents’ professional characteristics and ultrasound training levels. US: Ultrasound

	Frequency	Percentage
Residency Level		
Resident-1	40	34.5
Resident-2	34	29.3
Resident-3	22	19
Resident-4	20	17.2
Did Not Complete the US rotation		
No	19	16.4
Yes	97	83.6
	116	

Descriptive analysis of the residents’ perceptions about the usage, practice, and importance of POCUS is presented in Table 2. The findings showed that cardiac (including IVC) conditions were identified as the most anticipated scenarios to use POCUS (98.3%). Following closely, trauma cases were anticipated to require POCUS in 87.9%. Moreover, a significant majority of physicians (83.6%) anticipated the use of POCUS for procedural guidance, including central lines. Subsequently, hepato-biliary conditions were anticipated to use POCUS in 72.4% of cases, while 69.8% anticipated using it for DVT assessment. Moving down the list, a notable percentage of physicians (45.7% and 41.4%) anticipated that POCUS would be required for thoracic and aortic conditions, respectively. Pregnancy conditions were anticipated to require POCUS in 37.1% of cases, followed by soft tissue conditions at 25.9%. Finally, the least anticipated conditions for POCUS usage were renal, musculoskeletal, and ocular medical conditions, each with 19.8%, 8.6%, and 8.6% of physicians anticipating their usage respectively. When asked to indicate how many times, on average, they use an US probe on their patients per shift, the resulting findings showed that 19.8% of the residents use the US once per shift. However, the majority of them (75%) use US probes on their patients more than once per shift, and 5.2% use them on every patient when time allows them per shift. When asked about the importance of POCUS skills for EM residents, the findings revealed that 95.7% of respondents either agreed or strongly agreed that POCUS skills are indeed valuable for EM residents. Conversely,1.7% of respondents strongly disagreed and considered POCUS skills for EM residents as not important, while 2.6% remained neutral. When asked about the importance of practicing POCUS in the ED, the results showed that 55.2% strongly agreed and an additional 40.5% agreed that practicing POCUS was highly necessary in the ED. On the other hand, 1.7% strongly disagreed, while 2.6% remained undecided. However, when EM residents were asked to express their agreement regarding the importance of POCUS in their future EM practice. The results showed that 1.7% disagreed, while 6% were undecided. However, 35.3% agreed, and a substantial 56.3% strongly agreed with the contention that POCUS would be an important part of their future EM practice. Lastly, concerning the significance of POCUS availability when seeking employment, the results revealed that 31% agreed, and a significant majority of 48.3% strongly agreed that POCUS availability was crucial for their future job prospects. Conversely, 3.4% strongly disagreed and 2.6% disagreed that POCUS availability was crucial for their future job prospects. On the other hand, 14.7% were neutral. 

**Table 2:** Descriptive analysis of the residents’ perceptions about the usage, practice, and importance of POCUS. POCUS: Point-of-care ultrasound; DVT: deep vein thrombosis; IVC: inferior vena cava; US: ultrasound; ED: emergency department; EM: emergency medicine

	Frequency	Percentage
Which of the following indications do you anticipate using POCUS as an attending physician?		
Musculoskeletal	10	8.6
Ocular	10	8.6
Soft tissue	30	25.9
Pregnancy	43	37.1
Hepato-biliary	84	72.4
Procedural guidance (including central lines)	97	83.6
Trauma	102	87.9
DVT	81	69.8
Cardiac (including IVC)	114	98.3
Thoracic/lung	53	45.7
Aortic	48	41.4
Renal	23	19.8
On average, how many times per shift do you put an US probe on a patient?		
Once	23	19.8
More than one time	87	75
On every patient if time allows	6	5.2
POCUS is an important skill for residents to learn		
Strongly Disagree	2	1.7
Neutral	3	2.6
Agree	29	25
Strongly Agree	82	70.7
POCUS is an important skill to practice in our ED		
Strongly Disagree	2	1.7
Neutral	3	2.6
Agree	47	40.5
Strongly Agree	64	55.2
POCUS will be an important part of my future EM practice		
Strongly Disagree	2	1.7
Neutral	7	6
Agree	41	35.3
Strongly Agree	66	56.9
POCUS availability will be important for me when I look for a job		
Strongly Disagree	4	3.4
Disagree	3	2.6
Neutral	17	14.7
Agree	36	31
Strongly Agree	56	48.3

Descriptive analysis of the residents’ perceived barriers to POCUS performance during their daily clinical practice is presented in Table 3. The findings showed that the foremost barrier, as reported by physicians, was the lack of time available for conducting POCUS examinations (mean=4.16±1.02). Following this, physicians identified the time required to complete a full exam as the second most significant barrier. Additionally, difficulties in locating the medical US machine and obtaining lubricant gel were cited as notable obstacles. Subsequent barriers included challenges in utilizing results for documentation, a lack of knowledge regarding the credentials of attending physicians and struggles with understanding the machine's operating system. Moreover, medical residents identified considerations such as patients refusing USs (mean=1.67±1.02) and viewing US assessments as beyond their medical scope (mean=1.60±1.02) and as the least perceived barriers. Furthermore, the presence of the US radiology department, which is often readily available, was considered less obstructive.

**Table 3:** Descriptive analysis of the residents perceived barriers to POCUS performance during their daily clinical practice. SD: Standard deviation; POCUS: point-of-care ultrasound; US: ultrasound

	Mean	SD	Rank
Available time to start an exam	4.16	1.02	1
Time to complete/optimize a full exam	3.85	1.11	2
Inability to use in the results in documentation	3.69	1.26	5
Not knowing if your attending is credentialed	3.08	1.23	6
Difficult to figure out operating system	2.78	1.21	7
Can’t find the US machine	3.8	1.35	3
Can’t find the gel	3.72	1.36	4
The machine is out of space	2.72	1.27	8
Radiology US too readily available	2.28	1.19	9
Patient refusal	1.67	1.02	10
You don’t see it as within your scope of practice	1.60	1.02	11

Descriptive analysis of the residents’ perceived possible facilitators to POCUS usage during their daily clinical practice is presented in Table 4. The findings revealed that the top perceived facilitator for using POCUS among resident physicians was the support from attending physicians during shifts for decision-making. This was closely followed by the availability of clear guidelines for charting findings and the possibility of incentives for completing and submitting US scans by physicians. Furthermore, these top perceived facilitators were supplemented by the implementation of location trackers for the US machines due to their tendency to be misplaced within the hospital. Additionally, physicians believed that organizing conferences for attendance credits could enhance POCUS usage. The least agreed upon facilitators included displaying attending physicians' US certifications for using the US machines, hosting a celebratory dinner for residents with top submitted US scans, and showcasing attending physicians' US certificates in the hospital software. 

**Table 4:** Descriptive analysis of the residents’ perceived possible facilitators to POCUS usage during their daily clinical practice. SD: Standard deviation; POCUS: point-of-care ultrasound; US: ultrasound

	Mean	SD	Rank
Some kind of incentive for SUBMITTED complete scans	3.90	1.25	3
Attendings supporting POCUS on shift for medical decision making	3.98	0.83	1
Displaying attending US certifications in the hospital software	3.66	1.19	6
Displaying attending US certifications on the US machine	3.59	1.07	8
Providing clear guidelines on charting	3.95	1.05	2
Conference attendance credits	3.77	1.04	5
Class dinner for residents with most submitted scans	3.60	1.31	7
Implementing location trackers on machines	3.78	1.12	4

The results of the multivariable linear regression analysis examining residents' mean perceived overall barriers to POCUS usage during daily practice are presented in Table 5. The multivariable linear regression analysis examined several factors influencing the residents' mean perceived overall barrier to using POCUS in their daily practice. The multivariate analysis showed that the EM resident training level did not correlate significantly with their mean perceived POCUS machine use barriers score and p-value=0.632. But physicians who had completed their US rotation had perceived significantly higher barriers score compared to those who had not completed their medical residency US rotation on average, beta coefficient=0.40 and p-value=0.014; hence the medical residents who had previous exposure to the US machines during their rotations perceived higher barriers to US usage compared to those who had no previous exposure to those machines. Also, the findings showed that the physicians’ belief in the facilitating effect of incentives for using POCUS had correlated positively and significantly with their mean perceived barriers to POCUS usage score, beta coefficient=0.15, and p-value=.005, greater belief in incentives predicts significantly more perceived barriers to using the POCUS machines. Also, the physicians’ agreement level with displaying the attending US certificates on US machines as a facilitator had correlated significantly and positively with their mean perceived POCUS usage barriers score, beta coefficient=0.18, and p-value=0.005, therefore as physicians’ belief in the facilitator effect for displaying the attending US certifications on the machines tended to rise their perceived barriers to using those machines tended to also rose. Furthermore, physicians who anticipated the use of POCUS for DVT assessment had significantly lower mean perceived POCUS machine use barriers score compared to those who did not anticipate using the POCUS for DVT assessment, beta coefficient=-0.55, and p-value<0.001. Moreover, the physicians who believed that POCUS usage is anticipated for aortic assessment had significantly lower mean perceived POCUS use barriers score compared to those who did not anticipate the usage of POCUS machines for patient aortic assessment on average, beta coefficient=-0.24, and p-value=0.050.

**Table 5:** Multivariable linear regression analysis of the residents mean perceived overall barrier to POCUS usage during daily practice. POCUS: Point-of-care ultrasound; US: ultrasound; CI: confidence interval; DVT: deep vein thrombosis

	Unstandardized Coefficients	95.0% CI for Beta Coefficient		
		Lower Bound	Upper Bound	p-value
(Constant)	1.88	1.30	2.46	<0.001
Training level	0.03	-0.08	0.14	0.632
US rotation completed: Yes Vs. No	0.40	0.08	0.71	0.014
Believes some incentive for submitted complete scans enhances US usage	0.15	0.05	0.26	0.005
Believes Displaying attending US certifications on the US machine enhances the US usage	0.18	0.06	0.31	0.005
Use of POCUS For DVT assessment	-0.55	-0.80	-0.31	<0.001
Use of POCUS for Aortic assessment	-0.24	-0.48	0.00	0.050
Dependent Outcome variable: Overall mean perceived barriers to using the POCUS machine at workplace. Model R=.4, model R-squared=0.2.				

## Discussion

In this study, we investigated the perceptions and experiences of EM residents regarding the usage, practice, and importance of POCUS in their daily clinical practice. Our findings shed light on several significant aspects that contribute to the understanding of residents' attitudes toward POCUS and the barriers and facilitators they encounter in its implementation.

Our study revealed that a significant majority (83.6%) of EM residents had completed their US rotation, highlighting a growing emphasis on US training in residency programs. This underscores the recognition of POCUS as an essential skill set for future emergency physicians. Previous research by Rathbun et al. (2023), Kotagal et al. (2015), and El-Hussein et al. (2022) has similarly emphasized the increasing importance of US training within residency programs, aligning with our findings [25-27].

Moreover, our study revealed a positive attitude toward the importance of POCUS skills among residents, with a vast majority (70.7%) strongly agreeing on its significance. Similarly, a substantial proportion (55.2%) acknowledged the importance of POCUS in ED practice, indicating a widespread recognition of its clinical utility. These findings echo those of Oh et al. (2021) and Thomas et al. (2017), which have emphasized the value of POCUS in enhancing diagnostic accuracy and patient care in emergency settings [28,29].

Interestingly, residents also expressed their anticipation of POCUS playing a significant role in their future EM practice, with over half (56.9%) strongly agreeing on its importance. This forward-looking perspective underscores the need for robust training programs that adequately prepare residents for the evolving landscape of EM, where POCUS is poised to play a pivotal role. This aligns with the findings of Souleymane et al. (2021), which have highlighted the increasing demand for US skills among practicing emergency physicians [30].

Despite the recognition of POCUS's importance, our study identified several barriers hindering its widespread adoption in clinical practice. Chief among these barriers were time constraints for starting and completing a full exam and logistical challenges, such as locating US machines and navigating the documentation process. Addressing these barriers requires a multifaceted approach, including workflow optimization, technological enhancements, and targeted educational interventions. Several studies demonstrated multiple obstacles facing physicians in EM to use US in the shift. In a recent cross-sectional study in Australia, 52 EM trainees reported that time limitations, lack of knowledge on which image to capture, how to label the image, and low confidence regarding their own scan as the common barriers facing US use [31]. Moreover, the time limitation is not unique to the study by Elsayed et al., previous studies mentioned the same issue among their medical practitioners [21,32-35]. On the other hand, a lack of training was reported extensively in previous observational studies. Williams et al., through a cross-sectional study involving more than 100 chiefs of different specialties, stated that lack of training and even lack of protected time for training is the most commonly reported barrier in regard to POCUS usage [16]. Furthermore, there have been numerous international studies to report that training is not sufficient [21,36-38].

Furthermore, the findings of our multivariable regression analysis shed light on important clinical implications regarding the perceived barriers to POCUS usage among EM residents. Firstly, the observation that physicians who completed their medical US rotation perceived higher barriers to using US machines compared to those who hadn't suggests a potential gap in the effectiveness of current training programs. This underscores the importance of evaluating and potentially refining the content and delivery of US training within residency programs to ensure adequate preparedness and confidence among residents in utilizing POCUS. Moreover, the correlation between beliefs in the facilitating effect of incentives and the display of attending US certifications with higher perceived barriers highlights the nuanced relationship between external motivators and residents' attitudes towards POCUS integration. This suggests that simply providing incentives or displaying certifications may not be sufficient to overcome perceived barriers and may even exacerbate them. Instead, efforts should focus on addressing underlying concerns and enhancing residents' understanding and confidence in POCUS through targeted interventions. Conversely, the finding that physicians anticipating POCUS use for specific assessments like DVT and Aortic Assessment tend to perceive fewer barriers suggests the potential benefits of targeted training and education tailored to specific clinical contexts. By emphasizing the relevance and utility of POCUS in specific clinical scenarios, such as diagnosing DVT and assessing aortic conditions, residency programs can potentially mitigate barriers and foster greater acceptance and integration of POCUS into routine practice.

This study can be evaluated in terms of its strengths and limitations. Strengths of the study include its comprehensive data collection method, which utilized a self-administered questionnaire covering various aspects of EM residents' attitudes, experiences, and perceptions towards POCUS. Additionally, the inclusion of all current EM residents in the Riyadh region ensured a representative sample, and rigorous questionnaire development, including content validity testing and a high Cronbach's alpha value, ensured the reliability of the data collected. Furthermore, the diverse participant pool from various training centers in Riyadh enhanced the generalizability of the findings. However, limitations include reliance on self-reported data, and cross-sectional design which may restrict generalizability and hinder the establishment of causality. Additionally, the number of responders were lower than aimed which could affect the statistical analysis of the study. Lastly, potential confounders and the study's limited scope of analysis, particularly regarding institutional factors and patient outcomes, should be considered when interpreting the results.

## Conclusions

In conclusion, our study contributes to the growing body of literature on POCUS utilization among EM residents. By identifying key perceptions, barriers, and influencing factors, we aim to inform future educational initiatives and institutional policies aimed at optimizing POCUS training and utilization in EM residency programs. Ultimately, our findings underscore the importance of fostering a culture of innovation and continuous learning to ensure the effective integration of POCUS into clinical practice, thereby improving patient care outcomes in the ED.

## Appendices

**Table 6:** Descriptive analysis of the residents’ professional characteristics and ultrasound training levels. US: Ultrasound

	Frequency	Percentage
Residency Level		
Resident-1	40	34.5
Resident-2	34	29.3
Resident-3	22	19
Resident-4	20	17.2
Did Not Complete the US rotation		
No	19	16.4
Yes	97	83.6
	116	

**Table 7:** Descriptive analysis of the residents’ perceptions about the usage, practice and importance of POCUS. POCUS: Point-of-care ultrasound; DVT: deep vein thrombosis; IVC: inferior vena cava; US: ultrasound; ED: emergency department; EM: emergency medicine

	Frequency	Percentage
Which of the following indications do you anticipate using POCUS as an attending physician?		
Musculoskeletal	10	8.6
Ocular	10	8.6
Soft tissue	30	25.9
Pregnancy	43	37.1
Hepato-Biliary	84	72.4
Procedural guidance (including central lines)	97	83.6
Trauma	102	87.9
DVT	81	69.8
Cardiac (including IVC)	114	98.3
Thoracic/lung	53	45.7
Aortic	48	41.4
Renal	23	19.8
On average, how many times per shift do you put an US probe on a patient?		
Once	23	19.8
More than one time	87	75
On every patient if time allows	6	5.2
POCUS is an important skill for residents to learn		
Strongly Disagree	2	1.7
Neutral	3	2.6
Agree	29	25
Strongly Agree	82	70.7
POCUS is an important skill to practice in our ED		
Strongly Disagree	2	1.7
Neutral	3	2.6
Agree	47	40.5
Strongly Agree	64	55.2
POCUS will be an important part of my future EM practice		
Strongly Disagree	2	1.7
Neutral	7	6
Agree	41	35.3
Strongly Agree	66	56.9
POCUS availability will be important for me when I look for a job		
Strongly Disagree	4	3.4
Disagree	3	2.6
Neutral	17	14.7
Agree	36	31
Strongly Agree	56	48.3

**Table 8:** Descriptive analysis of the residents perceived barriers to POCUS performance during their daily clinical practice. SD: Standard deviation; POCUS: point-of-care ultrasound; US: ultrasound

	Mean	SD	Rank
Available time to start an exam	4.16	1.02	1
Time to complete/optimize a full exam	3.85	1.11	2
Inability to use in the results in documentation	3.69	1.26	5
Not knowing if your attending is credentialed	3.08	1.23	6
Difficult to figure out operating system	2.78	1.21	7
Can’t find the US machine	3.8	1.35	3
Can’t find the gel	3.72	1.36	4
The machine is out of space	2.72	1.27	8
Radiology US too readily available	2.28	1.19	9
Patient refusal	1.67	1.02	10
You don’t see it as within your scope of practice	1.60	1.02	11

**Table 9:** Descriptive analysis of the residents’ perceived possible facilitators to POCUS usage during their daily clinical practice. SD: Standard deviation; POCUS: point-of-care ultrasound; US: ultrasound

	Mean	SD	Rank
Some kind of incentive for SUBMITTED complete scans	3.90	1.25	3
Attendings supporting POCUS on shift for medical decision making	3.98	0.83	1
Displaying attending US certifications in the hospital software	3.66	1.19	6
Displaying attending US certifications on the US machine	3.59	1.07	8
Providing Clear guidelines on charting	3.95	1.05	2
Conference attendance credits	3.77	1.04	5
Class dinner for residents with most submitted scans	3.60	1.31	7
Implementing Location trackers on machines	3.78	1.12	4

**Table 10:** Multivariable linear regression analysis of the residents mean perceived overall barrier to POCUS usage during daily practice. POCUS: Point-of-care ultrasound; US: ultrasound; CI: confidence interval; DVT: deep vein thrombosis

	Unstandardized Coefficients	95.0% CI for Beta coefficient		
		Lower Bound	Upper Bound	p-value
(Constant)	1.88	1.30	2.46	<0.001
Training Level	0.03	-0.08	0.14	0.632
US rotation completed: Yes Vs. No	0.40	0.08	0.71	0.014
Believes some incentive for submitted complete scans enhances US usage	0.15	0.05	0.26	0.005
Believes Displaying attending US certifications on the US machine enhances the US usage	0.18	0.06	0.31	0.005
Use of POCUS For DVT assessment	-0.55	-0.80	-0.31	<0.001
Use of POCUS for Aortic assessment	-0.24	-0.48	0.00	0.050
Dependent Outcome variable: Overall mean perceived barriers to using the POCUS machine at workplace. Model R=.4, model R-squared=0.2.				

**Table 11:** Level of training. US: Ultrasound

What Is Your Level of Training?	
A. 1st year, completed US rotation	
B. 1st year, not yet completed US rotation	
C. 2nd Year	
D. 3rd Year	
E. 4th year	
On average, how many times per shift do you put an US probe on a patient?	
A. Never	
B. Once	
C. More than once	
D. On every patient	

**Table 12:** How do you feel about point-of-care ultrasound. POCUS: Point-of-care ultrasound; ED: emergency department; EM: emergency medicine

	Strongly Disagree	Somewhat Disagree	Neutral	Somewhat Agree	Strongly Agree
POCUS is an important skill for residents to learn					
POCUS is an important skill to practice in our ED					
POCUS will be an important part of my future EM practice					
POCUS availability will be important for me when I look for a job					

**Table 13:** Indications to use ultrasound. US: Ultrasound; DVT: deep vein thrombosis; IVC: inferior vena cava; MSK: musculoskeletal

Which of the Following Indications Do You Anticipate Using as an Attending?	
Cardiac (including IVC)	
Thoracic/lung	
Aortic	
Renal	
DVT	
Hepatobiliary	
Soft tissue/MSK	
Ocular	
Pregnancy	
Procedural guidance (including central lines)	

**Table 14:** Please rate the following barriers to performing POCUS. US: Ultrasound; POCUS: point-of-care ultrasound

	Not a Barrier	Slight Barrier	Moderate Barrier	Significant Barrier	Extreme Barrier
Available time to start an exam					
Time to complete/optimize a full exam					
Inability to use in the results in documentation					
Not knowing if your attending is credentialed					
Difficult to figure out the system					
Can’t find the US machine					
Can’t find the gel					
The machine is out of space					
Radiology US too readily available					
Patient refusal					
You don’t see it as within your scope of practice					

**Table 15:** How likely are the following interventions to get you to do POCUS on shift. POCUS: Point-of-care ultrasound; US: ultrasound

	Not Likely	Somewhat Unlikely	Neutral	Somewhat Likely	Very Likely
Some kind of incentive for SUBMITTED complete scans					
Attendings supporting POCUS on shift for medical decision					
Displaying attending US certifications in the system					
Displaying attending US certifications on the US machine					
Clear guidelines on charting					
Conference attendance credits					
Class dinner for class with most submitted scans					
Location trackers on machines					
